# Effects of CEO Identity on Non-family Managers’ Pay Dispersion in Family Firms: A Social Comparison Perspective

**DOI:** 10.3389/fpsyg.2021.683011

**Published:** 2021-10-01

**Authors:** Wei Zhang, Ling Chen, Jian-an Zhu

**Affiliations:** ^1^School of Economics, Zhejiang University, Hangzhou, China; ^2^School of Management, Zhejiang University, Hangzhou, China; ^3^Business School, Zhejiang University City College, Hangzhou, China

**Keywords:** social comparison theory, family firm, non-family managers, TMT pay dispersion, CEO tenure, marketization index

## Abstract

The fairness of compensation has been a prominent focus for non-family managers, and pay dispersion, which reflects compensation fairness, has attracted much attention from scholars. Based on social comparison theory, this study investigates the factors that affect the pay dispersion between CEO and non-family managers. In family firms, the role of CEO, which is central in corporate governance, can be filled by either a family or a non-family member. This study provides insights into how the identity of the CEO affects pay dispersion and investigates the moderating effects of CEO tenure and institutional environment. Using the data of Chinese listed family firms from 2009 to 2015, the results show that the presence of non-family CEOs could decrease the pay dispersion between CEO and non-family managers. Empirical evidence also supports that the negative relationship between CEO identity and pay dispersion weakens when CEO tenure increases and the institutional environment matures.

## Introduction

The continuous pursuit of sustainable development makes family firms compete fiercely for talent, and recruiting non-family managers often becomes a necessity. Considering family firms, the top management teams are often composed of both family and non-family managers, and the characteristics of the two types of managers have been explored by various studies (Chrisman et al., 2014; Tabor et al., 2018). Family managers can highlight family characteristics and consolidate family control (Bach and Serrano-Velarde, 2015), generate greater job security (Cai et al., 2010; Luo and Chung, 2013), and exhibit more emotional attachment to the firm. Conversely, non-family managers are generally selected from the competitive human resource market, and most have received formal education and training (Chrisman et al., 2014). These individuals are more available for the managerial positions than the limited number of family members (Fang et al., 2021). Therefore, to attract and motivate non-family managers to act in the best interests of the holding family, one possible action involves setting effective and fair incentive structures. Pay dispersion, as a manifestation of the fairness of incentive plans (Ding et al., 2009), could have implications for the working relationship of top management teams and became a critical human resource and corporate governance issue to be considered. Therefore, this study focused on the fairness of managerial compensation and investigated the pay dispersion among non-family managers.

Since managers have attached more importance to the fairness of compensation, factors that could affect the pay dispersion became a key issue discussed by scholars. Considering that executive compensation is mostly determined by the board of directors, most studies have tried to explore the influencing factors based on the attributes of the board of directors, such as board seat (Fredrickson et al., 2010) and ownership (Connelly et al., 2016; Keppeler and Papenfuß, 2021) on managerial pay dispersion. There are also studies examining how managerial attributes influence pay dispersion, such as managerial political ideologies (Chin and Semadeni, 2017), and morale effects (Breza et al., 2018). However, what cannot be neglected, as the leader of top management teams, the opinions of the CEO will have a degree of impact on the executive compensation arrangement. In addition, in family firms, the identity of the CEO could be a source of family firm heterogeneity (Barontini and Bozzi, 2018). The appointment of a family or non-family CEO could alter the holding families’ propensity to make decisions in firm governance and operation. Although CEO identity is a common corporate governance form in family firms, the effect of how CEO identity influences the pay dispersion has not received enough attention. Thus it is important to investigate whether CEO identity impacts the compensation arrangement for non-family managers and its implications for pay dispersion.

Although non-family managers have strengths such as competence and managerial experience, these attributes also put them in a similar and competitive status, which makes non-family managers likely referents for one another, and social comparison often occurs at the same time (Festinger, 1954). Compensation, as an initial symbol of a manager’s human capital value and social status (Main et al., 1993), becomes an important object of social comparison. Social comparison theory states that individuals focus on comparing their compensation to determine whether they receive fair treatment (Festinger, 1954; Fredrickson et al., 2010), and higher pay dispersion could lead to lower firm performance (Jaskiewicz et al., 2017; Patel et al., 2018; Zhang et al., 2020), or increased managerial turnover (Ridge et al., 2017; Kacperczyk and Balachandran, 2018). However, under the framework of social comparison theory, few studies have focussed on the factors that influence managerial pay dispersion in family firms. Therefore, our work adopts a social comparison perspective to investigate how CEO identity influences non-family managers’ pay dispersion in Chinese family firms.

The importance of CEO identity is also highlighted through society’s perceptions of individual competence and environmental informal rules. Hence, the influence of CEO identity on pay dispersion should also consider the moderating effects of certain competence and environmental factors. For competence factors, CEO tenure is an important indicator to identify a CEO’s competence and experience (Chen et al., 2019). A more capable CEO will be less likely to emphasize the importance of identity, which will decrease the comparison tendency among non-family managers. In addition, environmental factors like institutional environment should also be considered. A more standardized institutional environment leads to more attention on work efficiency and fair competition, which will lead to less focus on social comparisons, and less emphasis on the importance of CEO identity. Therefore, theories that seek to increase our understanding of how CEO identity influences pay dispersion must consider more fully the moderating effects of CEO tenure and institutional environment.

Using data from Chinese listed family firms from 2009 to 2015, we investigated the antecedent factors that could influence managerial pay dispersion. This study attempts to answer two related questions. (1) How does non-family CEO identity influence the pay dispersion between CEO and non-family managers? (2) How do CEO tenure and institutional environment moderate the relationship between CEO identity and pay dispersion?

This study contributes to the prior literature in several ways. First, our paper extends the recent work on the influencing factors of managerial pay dispersion in family firms. Under the framework of social comparison theory, our paper identifies CEO identity as an important influencing factor of managerial pay dispersion, and explores how CEO identity influences pay dispersion between CEO and non-family managers in family firms. Second, our work incorporates CEO identity into the analytical framework of social comparison theory, enriching its applicability. Even though maintaining pay fairness is of great significance, the current research had not yet indicated arrangements on how to reduce pay dispersion under the framework of social comparison theory. Hence, this paper incorporates CEO identity into the analytical framework of social comparison theory and finds an effective way to deal with pay dispersion. Third, our paper makes an in-depth exploration of social comparison theory and finds factors that could affect the social comparison tendency. As a kind of individual subjective psychological feeling, social comparison can also be affected by some external factors, like individual competence and external informal rules. Hence, our paper highlights CEO tenure and institutional environment as moderators to represent how individual competence and external rules affect the social comparison tendency. Also, our paper provides practical implications: our work adopts a reference on how to motivate non-family managers effectively, and the results show that non-family managers are highly sensitive to the fairness of compensation. In addition, the compensation arrangement should also consider CEO’s opinion.

The remainder of the paper is organized as follows. Section “Theoretical Background” presents a review of literature on social comparison theory and managerial pay dispersion, section “Hypotheses Development” puts forward the development of our hypotheses, section “Methodology” explains the model, data collection, and measurement of variables, section “Empirical Results” explains the statistical methods and empirical results, section “Robustness Tests” presents the robustness tests, and section “Discussion” discusses the implications and limitations of the research and provides suggestions for future research. “Conclusion” summarizes the results and implications of the research.

## Theoretical Background

### Social Comparison Theory

Social comparison theory was derived from the field of social psychology. As a general and fundamental feature of social life, Festinger (1954) noticed the phenomenon of social comparison, which showed that individuals had a “drive” to evaluate their opinions and abilities. The primary motive of social comparison is obtaining information from others, and then evaluating oneself. Social comparison theory argues that individuals have an intrinsic need to evaluate and compare their abilities and opinions with others within the same group or even other groups, and determine whether they receive fair treatment. Researchers have identified three motives of social comparison: evaluation, enhancement, and improvement (Taylor et al., 1996), which is also the process of social comparison. Furthermore, social comparison theory argues that the greater the difference in an individual’s ability, status, and opinion, the weaker the social comparison tendency. That is, individuals commonly compare themselves with others with whom they have attributes in common, such as position, demographic characteristics, or ability (Festinger, 1954; Gerber et al., 2018).

In recent studies, social comparison always occurs within firms when managers or employees compare with their “referents” (Gartenberg and Wulf, 2017). In family firms, non-family managers bring professional knowledge and management experience to the firm. As professional personnel who are selected from the competitive human resource pool, these non-family managers are likely to be achievement-oriented, compensation-sensitive, and power-seeking (Hiebl and Li, 2020). These similar attributes led them highly competitive with each other and made them particularly prone to make social comparisons (Tariq et al., 2021). Compensation, as an initial symbol of a manager’s human capital value and social status (Main et al., 1993), has naturally become an important object of social comparison. Non-family managers are likely to engage in social comparisons to compare their compensation to judge whether they are being treated fairly. If they receive far less compensation than their fellows, in other words, the pay dispersion is large, this will lead to a feeling of inequity. The assessment of inequity can lead to a feeling of injustice and jealousy, which in turn may reduce team cohesion and decrease job satisfaction (Collischon and Eberl, 2020), and even lower firm performance (Patel et al., 2018; Zhang et al., 2020). Conversely, if managers get relatively equal compensation with others, these managers may generate a perception of fair treatment, which in turn improves team collaboration and firm performance. Consequently, the comparative attributes of non-family managers have made social comparison theory beneficial in explaining executive pay dispersion issues. Most recent research on social comparison and pay dispersion focuses on how pay dispersion affects firm behavior, however, our work tries to adopt a social comparison perspective to investigate factors that could influence non-family managers’ pay dispersion in Chinese family firms.

### Managerial Pay Dispersion

According to Siegel and Hambrick (2005)’s work, managerial pay dispersion falls into two categories: vertical pay dispersion and horizontal pay dispersion. Vertical pay dispersion refers to the pay gap between different executive hierarchical levels, like the compensation differences between CEO and vice managers. Horizontal pay dispersion refers to the pay differences among managers within the same hierarchical level, like the compensation differences among all vice managers. In this study, we paid close attention to the group of non-family managers in family firms and focused on vertical pay dispersion between CEO and non-family managers.

By establishing a general view of managerial pay dispersion research, studies on how managerial pay dispersion influences firm behavior such as firm performance (Jaskiewicz et al., 2017; Patel et al., 2018; Zhang et al., 2020), executive turnover (Ridge et al., 2017), and firm innovation (Yanadori and Cui, 2013; Amore and Failla, 2020) have been well researched. For firm performance, Jaskiewicz et al. (2017) investigated this issue in family firms and found that pay dispersion among non-CEO top management team members could harm firm performance. For firm innovation research, Amore and Failla (2020) state that the variable pay dispersion leads to higher innovation. On the other hand, the larger dispersion in fixed pay leads to lower innovation. For managerial turnover research, Kacperczyk and Balachandran (2018) gave us a reference that vertical and horizontal pay dispersion led to different answers, that is, the horizontal wage comparisons could increase cross-firm turnover because these managers induce inequity concerns, but vertical wage comparisons could decrease the turnover across firms because they enhance self-motivation.

Given the fact that managerial pay dispersion can influence firm behavior, then a logical next question occurs: are there any antecedent factors that could affect the level of pay dispersion? Establishing a general view on the influencing factors of pay dispersion, since executive compensation is mostly determined by the board of directors, most researches have tried to explore the influencing factors based on the board of directors, such as board seat (Fredrickson et al., 2010) and ownership (Connelly et al., 2016; Keppeler and Papenfuß, 2021). Fredrickson et al. (2010) found that there was even an inversed *U* shape relationship between the proportion of top managers team who are also board members and the level of pay dispersion. Keppeler and Papenfuß (2021) stated that the ownership publicness could affect vertical pay dispersion, and the relationship was moderated by firm size. There are also researches examining how managerial attributes influence pay dispersion, such as managerial political ideologies (Chin and Semadeni, 2017) and morale effects (Breza et al., 2018). Chin and Semadeni (2017) indicate that CEOs’ liberal ideologies could reduce pay dispersion both among non-CEO managers and between CEO and non-CEO managers. However, recent research has ignored one of the most basic characteristics of CEOs in family firms—CEO identity. The appointment of a family or non-family CEO could alter holding families’ propensity to make decisions in firm governance and operation. To address this, we adopt a social comparison perspective to investigate how CEO identity influences managerial pay dispersion in Chinese family firms.

## Hypotheses Development

**FIGURE 1:**
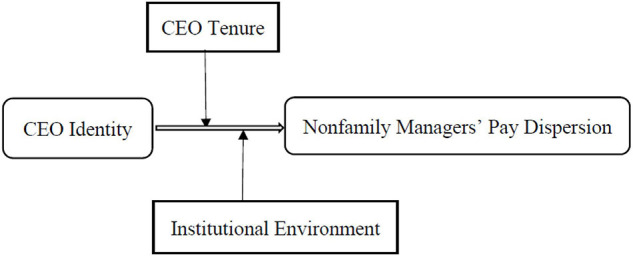
The theoretical model.

Our research focus is to understand the relationship between CEO identity and non-family managers’ pay dispersion, and the moderating effect of CEO tenure and institutional environment. Figure 1 is the theoretical framework of this research.

### Effects of CEO Identity on Non-family Managers’ Pay Dispersion

Social comparison theory argues that individuals commonly compare themselves to those with attributes in common, such as status, demographic characteristics, opinions, or ability (Gerber et al., 2018). In family firms, when the CEO is a family member, they would have grown up in the firm and been influenced by the surroundings of the firm and family. These family CEOs have experience and knowledge that are specific to the firm, and their history within the firm increases their understanding of the firm (Bach and Serrano-Velarde, 2015). As family members, these family CEOs could easily get firm ownership and management rights, so they are not only the owners of the firm but also the business operators who can gain management power (Kelleci et al., 2019). In contrast, non-family managers in family firms are treated as outside professional managers, and it is difficult for them to achieve the same level of power and resources compared to family CEOs get, which creates a “power distance” between family CEOs and non-family managers (Vandekerkhof et al., 2019). As “outsiders” of the firm, these non-family managers will recognize the inaccessibility of the “power distance,” so they choose to accept it, and lower their tendency to compare themselves with these family CEOs. However, with the development of the firm, an increasing number of family firms are recruiting non-family members to serve as CEOs (Fang et al., 2021). When non-family members hold the CEO position, both the CEO and non-family managers are professional personnel from the outside human resource market. Even if non-family managers served as CEOs, they couldn’t get as much ownership as family CEOs due to the necessity for family control. As “outsiders” of the firm, these non-family CEOs and non-family managers have relatively similar social status and power in family firms. According to social comparison theory, the equal social status and power render non-family managers prone to compare with other non-family managers, including non-family CEOs (Lazear and Rosen, 1981). When these non-family managers recognize that they have similar status and power as these non-family CEOs, it is natural for them to compare themselves with these non-family CEOs in all aspects, including compensation. They are particularly concerned with their compensation level as well as comparing that of non-family CEOs to determine whether they get fair treatment, and a large pay dispersion is likely to create a feeling of injustice and jealousy (Collischon and Eberl, 2020).

Although CEOs could not determine the compensation arrangement of the top management teams directly, as the implementer of the firm strategies they will still have an influence on such matters. In this way, to maintain the overall harmony and working enthusiasm of the top management teams, these non-family CEOs will advise the board of directors or the compensation committee to try to reduce the pay dispersion between the CEO and other non-family managers. As a result, to retain the human capital and social capital of these non-family managers, and by considering their calls for fairness, the board of directors or the compensation committee will also choose to set a fairer compensation structure to reduce the pay dispersion between the CEO and the non-family managers as much as possible. Hence, we put forward Hypothesis 1.

*Hypothesis 1: The presence of a non-family CEO is negatively associated with the pay dispersion between CEO and non-family managers.*

### The Relationship Between CEO Identity and Pay Dispersion With the Moderating Effect of CEO Tenure

Tenure is an important manifestation of the ability and experience of executives (Chen et al., 2019). Long-tenure CEOs have a higher likelihood of accumulating a large amount of tacit knowledge and possess a deep understanding of the firm’s current situation and future development, which is beneficial to obtaining competitive advantage (Pittino et al., 2018). Considering family firms, the presence of long-tenure non-family CEOs shows that they possess idiosyncratic knowledge that is compatible with family firms’ traits. They are more likely to invest a considerable amount of energy and time in reaching the required balance between the firm and family objectives and to share the same goals that these family firms are pursuing (Blumentritt et al., 2007). In addition, the long-tenure of a non-family CEO shows that their capability or human capital has been recognized by the holding family, which in turn shows increased trust from the family. In this way, the long-tenure non-family CEOs are more likely to obtain higher psychological ownership in family firms (Jiang et al., 2019). Under this circumstance, the non-family managers will recognize the value generated by these long-tenure CEOs, accept their contribution to the firm, and experience a feeling of admiration at the same time.

Furthermore, as long-tenure CEOs gain increased trust from the holding family, it is possible for them to get increased decision-making power at the same time (Phan, 1991). In addition, long-tenure CEOs usually possess higher voting shares, which contribute to their increased power on the board of directors (Darouichi et al., 2021). In this case, a long-tenure CEO possesses increased psychological ownership and higher decision-making power, which further causing a “power distance” between a long-tenure non-family CEO and other non-family managers. The “power distance” allows non-family managers to recognize the difference in ability and power between that of the long-tenure non-family CEOs, and at the same time accept the “power distance” due to the difference in decision-making power and psychological ownership, which could lead to a decreased tendency for social comparison. Even if the long-tenure non-family CEOs receive higher compensation than other non-family managers, these non-family managers will not generate a strong sense of unfairness, which means the pay dispersion between the two parties will not affect the perception of fairness. As a result, these arguments suggest that the presence of a long-tenure non-family CEO decreases the likelihood of social comparisons between the CEO and non-family managers, and even if there is a large pay dispersion between them, these non-family managers will not have strong feelings of inequity. Hence, we put forward Hypothesis 2.

*Hypothesis 2: CEO tenure moderates the relationship between CEO identity and pay dispersion, such that the hypothesized negative effect weakens as CEO tenure increases.*

### The Relationship Between CEO Identity and Pay Dispersion With the Moderating Effect of Institutional Environment

Williamson (2000) contended that the institutional environment is of great significance to organizational structure and behavior, and the maturity of the institutional environment provided policy support for firms’ sustainable development. Factors of the institutional environment include formal law systems, the financial market, and government policies are closely related to business operations. In the emerging market like China, due to the differences in resource endowment, geographical location, and regional policies in different regions, the maturity of institutional environments varies greatly even within a country. In China, the marketization level can reflect the maturity of an institutional environment (Wang et al., 2018). Even if government policies and laws are much the same across the country, the difference in institutional efficiency across regions has led to differences in corporate tax rates and resource expenditure (Greer and Doellgast, 2017). The imbalance in regional development also leads to the imbalance in marketization level.

A higher level of marketization creates a market environment that emphasizes formal contracts, rules, and firm efficiency, but not relational exchanges (Banalieva et al., 2015). With the improvement of the marketization process, formal laws, norms, and regulations have gradually become the source of institutional legitimacy for family firms’ survival and development. In regions with higher marketization levels, attracting external human capital and corporate investment (venture capital, strategic investors, etc.) will be more dependent on the standardization of corporate governance structure rather than simply on interpersonal networks (Xie, 2017). In areas with higher marketization levels, enterprises prefer to exhibit relatively better endowment of human capital, generate innovation and environmental protection, expand the recruiting of competent personnel and adopt a performance-based compensation system (Bin et al., 2020), which focuses managers more on their ability, as opposed to just comparing compensation. Numerous studies have shown that, in family firms, non-family managers have higher pay-performance sensitivity, which means that compensation for non-family managers is always set according to their performance (Michiels et al., 2013). In this situation, in areas with higher marketization levels, non-family managers’ compensation is always set based on their contribution and output to the firm, rather than blindly comparing their compensation level with others. The explicit performance-oriented compensation system enables non-family managers to focus more on their personal ability and performance, which naturally affects managers’ sense of fairness. At this time, even though non-family CEOs receive higher compensation due to their higher value of human capital, which may generate a large pay dispersion compared with other non-family managers, these non-family managers will accept the pay dispersion, and the tendency to compare compensations with each other is so weak that it would not affect the perception of fairness. Consequently, these arguments suggest that the social comparison tendency between the CEO and non-family managers will decrease as the institution environment matures, and even if there is a large pay dispersion between them, these non-family managers will not have strong feelings of inequity. Hence, we put forward Hypothesis 3.

*Hypothesis 3: Institutional environment moderates the relationship between CEO identity and pay dispersion, such that the hypothesized negative effect weakens as the institutional environment m*atures.

## Methodology

### The Model

We would like to test how CEO identity affects the pay dispersion between CEO and non-family managers, and the moderating effect of CEO tenure and institutional environment. The models were set as follows:


(1) $$PayGap_{it}=\beta_{0}+\beta_{1}NonFamCEO_{it}+\beta_{2}C_{it}+\beta_{3}Year_{it}+\beta_{4}Industry_{it}+\varepsilon_{it}$$

(2) $$PayGap_{it}=\beta_{0}+\beta_{1}NonFamCEO_{it}+\beta_{2}Tenure_{it}+\beta_{3}NonFamCEO_{it}\times Tenure_{it}+\beta_{4}C_{it}+\beta_{5}Year_{it}+\beta_{6}Industry_{it}+\varepsilon_{it}$$

(3) $$PayGap_{it}=\beta_{0}+\beta_{1}NonFamCEO_{it}+\beta_{2}Market_{it}+\beta_{3}NonFamCEO_{it}\times Market_{it}+\beta_{4}C_{it}+\beta_{5}Year_{it}+\beta_{6}Industry_{it}+\varepsilon_{it}$$

In Eq. (1), where *PayGap*_it_ indicates the dependent variable, is the pay dispersion between CEO and non-family managers; NonFamCEO_it_is the independent variable, which indicates the family or non-family identity of the CEO; *C*_*it*_ represents a set of control variables, including financial factors, managers’ background, firm size, firm age, etc.; Year_it_ and Industry_it_ indicate the dummy variables representing year and industry, respectively; and *ε*_*it*_ is the random interference item. In Eq. (2), we added the interaction term of CEO identity and CEO tenure to test the moderating effect of CEO tenure on the relationship between CEO identity and pay dispersion. Similarly, Eq. (3) added the interaction term of CEO identity and institutional environment to test the moderating effect of institutional environment on the relationship between CEO identity and pay dispersion.

### Data Sources and Variable Selection

The empirical data used in this study were derived from Chinese family firms listed on Shenzhen and Shanghai stock exchanges between 2009 and 2015. For data sources, we relied on the major dataset of China Stock Market and Accounting Research (CSMAR). The companies’ annual reports were also recorded to collect data on the identified firms. Data including managers’ identity, compensation, and some control variables like ROA (return on asset), financial leverage, etc. were obtained from the CSMAR database. The firms’ annual reports were collected from the official websites of Shenzhen and Shanghai stock exchanges. The annual reports could provide managers’ background information. The sample firms are all family firms. For the selection criteria of family firms, we adopted a threshold of 20% firm ownership by the family, as recommended by La Porta et al. (1999). Another criterion is that at least one family member, including persons related by blood connection or by marriage to the holding family, served as directors, shareholders, or managers (Anderson and Reeb, 2003). To ensure the reliability of the data, we excluded firms whose primary industry was financial services, because financial firms have different financial statement structures. Most of these firms have extraordinary debt ratios, which could lead to biased regression results.

Our dataset is an unbalanced panel data, for the following reasons. First, there were some samples in which managers were not paid by the firm or managers’ compensation information was not disclosed. To prevent missing values in the dataset, we excluded these samples at certain years. Second, we excluded samples in which the firms were sold, went bankrupt, or whose controller changed during our sample period, but still reserved these sample firms before they are sold or went bankrupt. Third, since our purpose is to examine the pay dispersion under social comparison theory, we suppose that a CEO could get more compensation than other managers, which means that the CEO’s compensation will be equal to or higher than non-family managers’ compensation, so we excluded samples in which the CEO got less compensation than non-family managers. For these reasons, we had an unbalanced panel including 255 firms representing 1,330 firm-year observations between 2009 and 2015.

However, variables like CEO and other managers’ family or non-family identity, and their baseline information like education background, age, compensation level, etc. could not be obtained directly from the database, so we manually distinguished and calculated these variables. The distinguishing and calculation process of the variables were as follows: first, to determine CEO identity, we downloaded the managers’ resumes from the CSMAR database, then searched their names online to determine whether they had a familial relationship with the holding family. If there was a familial relationship, we coded them as family CEOs, and others are coded as non-family CEOs. Second, besides CEO identity, we also distinguished other managers’ family or non-family identity in the same way and counted the number of non-family managers in the top management teams. Third, after calculating the number of non-family managers, we collected information on these managers’ age, education, and compensation, and calculated the pay dispersion between CEOs and non-family managers.

#### Independent Variable

CEO identity is the independent variable. According to Kelleci et al. (2019), CEO identity is a dummy variable that is distinguished by a non-family member holding a CEO position (value = 1) from a family member holding a CEO position (value = 0). Since CEO’s family or non-family identity could not be determined directly from the database, we manually distinguished the variable by seeking CEO resumes from the annual reports and by searching CEOs’ names online to determine whether they had a familial relationship with the holding family.

#### Dependent Variable

Pay dispersion, which represents the compensation gap between CEO and non-family managers, was the dependent variable. Pay dispersion (Pay Gap) was measured by a CEO’s cash compensation divided by the average cash compensation of all non-family managers (Siegel and Hambrick, 2005). These non-family managers were designated as second-level executives. In most cases, we assume that CEO would get more compensation than other second-level managers, so the pay dispersion is usually greater than 1 (value ≥ 1). The greater the value, the larger the pay dispersion between CEO and non-family managers (Siegel and Hambrick, 2005). There was another measurement of pay dispersion (Pay Gap 2), which was measured by the logarithmic value of a CEO’s compensation minus the average compensation of all non-family managers. The second measure was used in the robustness test.

#### Moderators

Two variables were utilized as moderators. The first was CEO tenure. According to the research of Chen et al. (2019), CEO tenure is defined as the number of years since the CEO took office. We searched for information about the year in which the manager took the position of CEO, and the variable was calculated by the current year minus the year when the manager took the position of CEO.

The second moderator was the institutional environment. The marketization level can represent the maturity of the institutional environment in China (Ruan et al., 2019). In our work, we used the “marketization index of China’s provinces” presented by Chinese economists Xiaolu Wang et al. (2018) to represent the maturity of the institutional environment in different regions. Since the policy of reform and opening-up began in 1978, the marketization level in China has been continuously increasing. However, the unbalanced regional economic development in China has led to wide differences in marketization levels in various regions. Wang, Fan, and their team have compiled a series of indices to represent different marketization levels for different provinces in China. The following five factors are used to calculate the marketization level: the relationship between government and market, the development of the non-state sector, the development of the product market, banking sector marketization, and the development of the legal environment (Wang et al., 2018). The index not only calculates the regional institutional development level of each province horizontally but also provides a series of indices annually to obtain a relatively complete set of panel data to measure the marketization process in different years and regions.

#### Control Variables

Consistent with the previous study (Jaskiewicz et al., 2017), we controlled for several possible determinants of pay dispersion. These variables fell into three categories: firm characteristics, managerial attributes, and corporate governance characteristics. For firm characteristics, we controlled for firm age, firm size, and firm performance. We defined firm size as the logarithm of total sales in the current year because managers in larger firms have a greater job variety than in smaller firms, which may result in different compensation levels among managers with different job classifications. Firm age is defined as the number of years since the firm was founded. Past firm performance was also controlled using the return on assets of the previous year because managers’ compensation is closely correlated with firm performance (Fredrickson et al., 2010). We controlled for ROA of the previous year (year*_*t*_*_–__1_) because managers’ compensation is always set based on firm performance of the previous year, rather than the current year. The financial leverage ratio, which is used as a measure of financial risk, was also controlled.

For the variables of managerial attributes, the traits of CEO and non-family managers may influence the level of pay dispersion (Fredrickson et al., 2010). First, we controlled for CEO age, average non-family managers’ age, CEO education, and average non-family managers’ education. These demographic factors could have an impact on executive compensation. Managers’ stockholdings are also a determinant of their compensation level, so we also controlled for CEO’s shareholding and non-family managers’ shareholding. Furthermore, we controlled for CEO duality. CEO duality took a value of “1” when the CEO holds the position of chairman of the board simultaneously.

Lastly, managerial pay dispersion is also likely to be affected by corporate governance characteristics. Thus, since the board of directors has the authority to determine managers’ compensation level, the board characteristics could affect the pay dispersion to some extent. In this way, we controlled for the size of the board and independent directors. Board size was measured by the number of board members. Independent directors were measured by the proportion of independent directors in the board of directors, which reflects board effectiveness.

In addition, we controlled for the industry using a dummy variable representing manufacturing sectors and other sectors, because there are a large proportion of Chinese family firms engaged in manufacturing sectors. Firms in manufacturing sectors were coded as “1” for each variable, and firms in other industries were coded as “0”. In addition, a series of dummy variables were used to measure the year in which the data were collected (2009 to 2015) to control for the possibility of periodic fluctuations. The variable summary is listed in Table 1.

**TABLE 1:** Variables summary.

**Category**	**Variables**	**Symbol**	**Measure**
Dependent variable	Non-family managers’ pay dispersion	Pay gap	Pay gap = CEO’s cash compensation/non-family managers’ average cash compensation.
Independent variable	CEO identity	Nonfam CEO	A binary variable; non-family members hold CEO position are coded as 1, otherwise 0.
Moderators	CEO tenure	Tenure	The number of years since CEO took office.
	Institutional environment	Market	The marketization index of China’s provinces presented by Wang et al. (2018)
Control variables	Firm age	Firm age	The number of years since the family firm was established.
	Firm size	Firm size	The natural logarithm of total sales at the end of each year.
	Firm performance	ROA	Return on asset of the previous year.
	Financial leverage ratio	Leverage	Financial leverage of the previous year.
	BETA	Beta	Beta at the end of the year.
	CEO age	CEO age	CEO’s age in the current year.
	Non-family managers’ age	TMT age	Average non-family managers’ age.
	Non-family managers’ education background	CEO edu	1 = Middle school; 2 = Junior college; 3 = Bachelor degree; 4 = Master degree; 5 = PhD.
	CEO’s education background	TMT edu	1 = Middle school; 2 = Junior college; 3 = Bachelor degree; 4 = Master degree; 5 = PhD.
	CEO’s shareholding	CEO share	Shareholding ratio of the CEO.
	Non-family manager’s shareholding	TMT share	Average shareholding ratio of non-family managers.
	CEO duality	CEO duality	A binary variable, coded 1 if the CEO also served as the chairman of the board of directors, otherwise 0.
	Board size	Board	The natural logarithm of number of board members.
	Independent directors ratio	Independent	The percentage of independent directors in the board of directors.
	Year	Year	Dummy variables, the sample spans from 2009 to 2015 (7 years), so we have 6 dummy variables of each year.
	Industry	Industry	A binary variable: firms from manufacturing sectors are coded 1, otherwise 0.

## Empirical Results

### Descriptive Statistics of the Variables

Table 2 presents the descriptive statistics of the variables. The average pay dispersion between the CEO and non-family managers was 1.811 with a maximum value of 12.05, indicating that the pay dispersion exists between CEO and non-family managers. For CEO identity, the value was 0.547, indicating that 54.7% of CEOs in the sample were non-family members. The average age of family firms in our sample was 16.73 years. Additionally, the average value of CEO tenure was 6.799, with a maximum value of 28. The mean value of CEO education and non-family managers’ education were 3.238 and 3.052, respectively, which indicated that CEOs had higher education levels than non-family managers.

**TABLE 2:** Descriptive statistics.

**Variables**	**Obs**	**Mean**	**Std. Dev.**	**Min**	**Max**
Pay gap	1,330	1.811	0.863	1.007	12.05
NonfamCEO	1,330	0.547	0.498	0	1
Market	1,330	7.974	1.485	−0.300	9.950
Tenure	1,330	6.799	5.336	1	28
Firm size	1,330	7.529	1.118	2.565	11.29
Firm age	1,330	16.73	7.463	3	63
ROA	1,330	0.0497	0.0592	−0.507	0.399
Leverage	1,330	3.170	7.300	0.079	132.0
Beta	1,330	1.035	0.199	0.191	1.743
CEO age	1,330	48.72	6.379	28	73
CEO edu	1,330	3.238	0.954	1	5
CEO duality	1,330	0.332	0.471	0	1
CEO share	1,330	0.081	0.148	0	0.691
TMT age	1,330	44.49	4.027	33.33	63
TMT edu	1,330	3.052	0.524	1.400	4.333
TMT share	1,330	0.011	0.0293	0	0.300
Board	1,330	2.123	0.169	1.386	2.708
Independent	1,330	0.371	0.053	0.200	0.667

### Correlation Analysis

The correlation matrix of variables is listed in Table 3. As indicated in the table, the correlation coefficient between CEO identity and pay dispersion is negatively significant (β = −0.101, *p* < 0.05). The data showed that the presence of a non-family CEO was negatively related to the pay dispersion between the CEO and non-family managers, which is consistent with Hypothesis 1. Combining the correlations among all these variables, we found that the correlation coefficients between independent variables and most control variables were small. VIF (variance inflation factor) could be an indicator of multicollinearity. VIF values higher than 10 indicate that these variables suffer from multicollinearity, and VIF values lower than 10 indicate that multicollinearity is not significant within these variables. In our model, the VIF values were all less than 1.8, indicating that multicollinearity is not a significant problem in our research.

**TABLE 3:** Correlation analysis (*P < 0.1, **P < 0.05, and ***P < 0.01).

**Variables**	**1**	**2**	**3**	**4**	**5**	**6**	**7**	**8**	**9**
1. Pay gap	1								
2. NonfamCEO	−0.101**	1							
3. Market	–0.034	–0.038	1						
4. Tenure	−0.055**	−0.398*	0.034	1					
5. Firm size	–0.035	0.007	–0.024	0.028	1				
6. Firm age	–0.043	0.053*	0.026	0.038	0.038	1			
7. ROA	–0.012	–0.001	−0.101***	0.019	0.191***	−0.059**	1		
8. Leverage	–0.013	0.001	0.061**	0.046	0.015	0.025	−0.161***	1	
9. Beta	–0.011	0.021	0.129***	0.018	−0.119***	0.029	−0.175***	0.048*	1
10. CEO age	0.074***	−0.097***	−0.095***	0.272***	–0.021	0.113***	0.038	0.063**	–0.019
11. CEO edu	0.027	–0.017	0.057**	0.086***	0.055*	−0.084***	0.056*	−0.054*	–0.007
12. CEO duality	0.067**	−0.528***	–0.004	0.298***	−0.082***	0.007	−0.055*	0.018	0.057**
13. CEO share	0.029	−0.443***	0.039	0.306***	−0.089***	−0.055*	0.048	−0.057**	0.052*
14. TMT age	–0.036	0.104***	–0.019	0.057**	0.086***	0.117***	0.052*	–0.015	−0.059**
15. TMT edu	−0.074***	0.039	−0.083***	0.055**	0.071**	0.036	0.028	–0.008	–0.027
16. TMT share	−0.087***	−0.072**	−0.096***	0.147***	–0.001	0.083***	0.081***	−0.048*	0.084***
17. Board	−0.051*	−0.137***	0.123***	0.358***	0.133***	0.115***	0.011	0.029	0.039
18. Independent	0.007	−0.131***	–0.006	0.165***	–0.008	0.029	0.019	–0.011	0.002

	**10**	**11**	**12**	**13**	**14**	**15**	**16**	**17**	**18**

10. CEO age	1								
11. CEO edu	−0.179***	1							
12. CEO duality	0.204***	−0.046*	1						
13. CEO share	0.098***	–0.042	0.514***	1					
14. TMT age	0.115***	–0.0078	–0.009	–0.018	1				
15. TMT Edu	0.021	0.260***	0.042	–0.039	−0.076**	1			
16. TMT share	0.027	–0.042	0.043	0.192**	–0.053	–0.008	1		
17. Board	0.095***	0.036	0.013	0.115**	−0.068**	0.133**	0.009	1	
18. Independent	0.092***	0.019	0.226**	0.215**	0.033	0.103**	−0.079**	0.070**	1

### Hypotheses Tests

In this study, STATA13.0 was used for data processing to test the hypotheses. Due to the multi-level nature of our theoretical model and data, the hypotheses were tested with the random-effects model. According to the previous family firm research (Cruz et al., 2014), the random-effects model is widely used in the researches on family firm corporate governance, because this method can reduce heteroscedasticity. Before estimating our model, the Hausman test was performed, and the result supported that of the random-effects model. Furthermore, to ensure the model’s consistency and validity, the data was processed as follows. First, to avoid the impact of outliers, Winsorize was applied at a 1% level for all continuous variables. Second, variables in the interaction items were centralized to avoid the influence of multicollinearity.

The regression results were shown in Table 4. In the first step, as a base model, only the moderator and control variables were included in Model 1. The results showed that the attributes of CEOs and non-family managers, such as their age and education, exerted significant effects on pay dispersion. The coefficients of CEO age (β = 0.015, *p* < 0.01) and CEO education (β = 0.144, *p* < 0.01) were significantly positive, which indicated that the presence of an older and higher educated CEO could lead to a higher level of pay dispersion between the CEO and non-family managers. Conversely, the coefficients of non-family managers’ age (β = −0.017, *p* < 0.05) and education (β = −0.205, *p* < 0.01) were significantly negative. The results showed that the presence of older and higher educated non-family managers is negatively related the pay dispersion. In step two, CEO identity (Nonfam CEO) was added as the independent variable in Model 2. After controlling for all these control variables, a CEO’s non-family identity is negatively related to the pay dispersion between the CEO and non-family managers (β = −0.255, *p* < 0.01), which suggested that the presence of a non-family CEO will decrease the pay dispersion between the CEO and non-family managers, and thus Hypothesis 1 was supported.

**TABLE 4:** Regression model results.

**Variables**	**Pay Gap**				
	**Model 1**	**Model 2**	**Model 3**	**Model 4**	**Model 5**
NonfamCEO		−0.255*** [0.086]	−0.210** [0.088]	−1.025*** [0.359]	−0.969*** [0.359]
NonfamCEO × Tenure			0.030** [0.015]		0.030** [0.015]
NonfamCEO × Market				0.096** [0.044]	0.095** [0.043]
Market	0.003 [0.028]	−0.002 [0.027]	−0.000 [0.027]	−0.068* [0.041]	−0.066 [0.041]
Tenure	−0.017** [0.008]	−0.024*** [0.008]	−0.036*** [0.010]	−0.023*** [0.008]	−0.035*** [0.010]
Firm age	0.000 [0.006]	0.001 [0.006]	0.001 [0.006]	−0.000 [0.006]	0.000 [0.006]
Firm size	0.026 [0.035]	0.019 [0.035]	0.018 [0.034]	0.017 [0.034]	0.016 [0.034]
ROA	−0.459 [0.461]	−0.487 [0.460]	−0.497 [0.459]	−0.525 [0.459]	−0.534 [0.458]
Leverage	−0.017 [0.022]	−0.016 [0.022]	−0.013 [0.022]	−0.013 [0.022]	−0.010 [0.022]
Beta	−0.022 [0.132]	−0.015 [0.132]	−0.002 [0.132]	−0.009 [0.131]	0.004 [0.131]
CEO age	0.015*** [0.005]	0.016*** [0.005]	0.015*** [0.005]	0.016*** [0.005]	0.015*** [0.005]
CEO edu	0.144*** [0.038]	0.139*** [0.038]	0.143*** [0.038]	0.138*** [0.038]	0.143*** [0.038]
CEO share	0.001 [0.003]	0.000 [0.003]	0.001 [0.003]	0.000 [0.003]	0.001 [0.003]
CEO duality	0.196** [0.082]	0.096 [0.088]	0.112 [0.089]	0.096 [0.088]	0.112 [0.088]
TMT age	−0.017** [0.008]	−0.015* [0.008]	−0.016** [0.008]	−0.014* [0.008]	−0.016* [0.008]
TMT edu	−0.205*** [0.070]	−0.185*** [0.070]	−0.182*** [0.070]	−0.177** [0.070]	−0.174** [0.070]
TMT share	−1.854 [1.349]	−1.763 [1.343]	−1.933 [1.344]	−1.851 [1.341]	−2.018 [1.342]
Board	0.004 [0.006]	0.004 [0.006]	0.006 [0.006]	0.005 [0.006]	0.006 [0.006]
Independent	0.528 [0.621]	0.579 [0.618]	0.665 [0.619]	0.525 [0.618]	0.610 [0.618]
Industry	Control	Control	Control	Control	Control
Year	Control	Control	Control	Control	Control
_cons	1.591** [0.664]	1.693** [0.662]	1.762*** [0.662]	2.227*** [0.703]	2.287*** [0.703]
Wald χ^2^	50.33	59.51	64.00	64.64	69.00
*N*	1,330	1,330	1,330	1,330	1,330

*p < 0.1; **p < 0.05; and ***p < 0.01.

Two moderators (CEO tenure and institutional environment) were entered in step three (Table 4, Models 3 and 4). Models 3 and 4 tested Hypothesis 2 and Hypothesis 3, respectively. The interaction terms between the independent variable and moderators were also included in this step. In Model 3, the interaction term of CEO identity and CEO tenure (NonfamCEO × Tenure) was added. The results showed that the coefficient of this interaction term was positively significant (β = 0.030, *p* < 0.05), which suggested that CEO tenure can moderate the relationship between CEO identity and pay dispersion, and the hypothesized negative effect weakens as CEO tenure increases. Hypothesis 2 was established. The interaction item of CEO identity and institutional environment (NonfamCEO × Market) was added into Model 4, and the coefficient of CEO identity and institutional environment was positive and significant (β = 0.096, *p* < 0.05). This result indicated that the institutional environment negatively moderated the relationship between CEO identity and pay dispersion, and the hypothesized negative effect weakens as the institutional environment matures, so Hypothesis 3 was also established. Furthermore, Model 5 is the full model, which means that the independent variable, two moderators, and all the control variables were included in this model. The results in Model 5 showed that all the hypotheses were supported.

**FIGURE 2:**
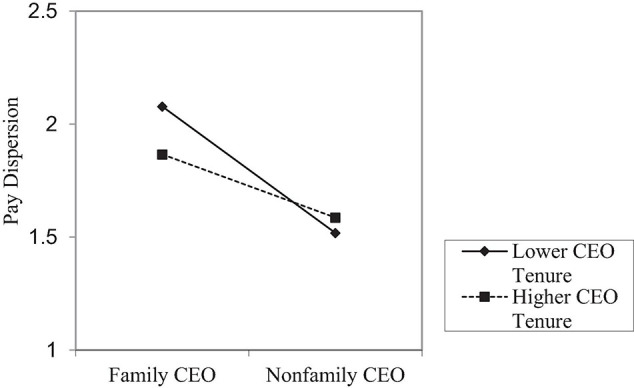
The moderation effect by CEO tenure.

**FIGURE 3:**
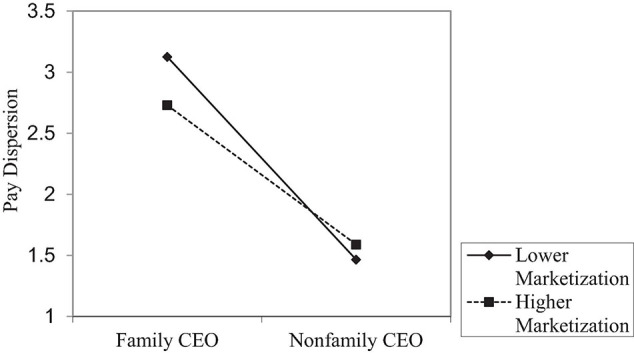
The moderation effect by marketization level.

To further facilitate the interpretation of the moderating mechanism, we plotted the moderating effects of CEO tenure and institutional environment in Figures 2, 3. As shown in Figure 2, the downward slope became much gentler when the CEO has longer tenure, which is consistent with Hypothesis 2. In support of Hypothesis 3, Figure 3 shows that the downward slope became much gentler when the marketization level is higher, which is also consistent with Hypothesis 3.

## Robustness Tests

We performed several additional tests to ensure the robustness of our results. First, we changed the measurement of the independent variable. The presence of a non-family CEO could reflect family firms attaching more importance to professionalization. In the robustness test, we use the percentage of non-family managers in top management teams to substitute the previous independent variable (Fang et al., 2021), because the percentage of non-family managers can also represent a concern for professional management in family firms. We ran the main effect and moderating effects again to show the robustness of the new independent variable, and the overall results were listed in Table 5. Compared with the results in Table 4, the statistical results in Table 5 were not remarkably different. To simplify the results, control variables were not listed in Table 5. In the first step, only the moderators and control variables were included in Model 1. In step two, the percentage of non-family managers (NonfamTMT Rate) was added as the independent variable in Model 2. The main effect of non-family managers’ percentage and pay dispersion was negatively significant (β = −0.867, *p* < 0.01). Similar to Table 4, two interaction variables were added to the model (Models 3 and 4, Table 5). In Model 3, the interaction of non-family managers’ percentage and CEO tenure (NonfamTMTRate × Tenure) was positive and significant (β = 0.080, *p* < 0.05). In Model 4, the interaction of non-family managers’ percentage and institutional environment (NonfamTMTRate × Market) was also positive and significant (β = 0.509, *p* < 0.01). Model 5 is included all the independent variables, moderators, and control variables, and the results in Table 5 are also significant.

**TABLE 5:** Robustness test: Substitute independent variable.

**Variables**	**Pay Gap**				
	**Model 1**	**Model 2**	**Model 3**	**Model 4**	**Model 5**
NonfamTMT rate		−0.867*** [0.243]	−0.558** [0.232]	−0.588*** [0.229]	−0.517** [0.231]
NonfamTMTRate × Tenure			0.080** [0.039]		0.089** [0.041]
NonfamTMTRate × Market				0.509*** [0.148]	0.529*** [0.148]
Tenure	[−0.017]**	−0.020*** [0.008]	−0.015*** [0.007]	−0.017*** [0.007]	−0.026** [0.026]
Market	−0.003 [0.028]	−0.002 [0.027]	−0.005 [0.026]	−0.026 [0.026]	−0.005 [0.026]
Control variables	Control	Control	Control	Control	Control
_cons	1.591 [0.664]	0.570 [1.121]	0.525 [1.121]	0.588 [1.116]	0.497 [1.117]
Wald χ^2^	63.73	63.73	72.82	80.99	86.75
*N*	1,330	1,330	1,330	1,330	1,330

*p < 0.1; **p < 0.05; and ***p < 0.01.

Second, we changed the measurement of the dependent variable. We used another measurement of pay dispersion between CEO and non-family managers as the new dependent variable (Pay Gap 2), which is measured by the logarithmic value of a CEO’s cash compensation minus the average cash compensation of all non-family managers [In (CEO compensation – the average compensation of non-family managers)]. The results were shown in Table 6. In Model 1, only moderators and control variables were added. Model 2 examined the main effect, which was significantly negative (β = −0.205, *p* < 0.05), which means Hypothesis 1 was also supported. Similar to Table 4, the interaction term of CEO identity and CEO tenure was added in the model (Model 3, Table 6), and the results are significantly positive (β = 0.030, *p* < 0.05). In Model 4, the interaction term of CEO identity and institutional environment was also significantly positive (β = 0.079, *p* < 0.1). Compared with the results in Table 4, the results shown in Table 6 did not show a remarkable difference. In all cases, we obtained results that were consistent with the main results reported above, and show that our conclusions are robust.

**TABLE 6:** Robustness test: Substitute dependent variable.

**Variables**	**Pay Gap 2**				
	**Model 1**	**Model 2**	**Model 3**	**Model 4**	**Model 5**
NonfamCEO		−0.205** [0.088]	−0.329*** [0.111]	−0.801** [0.356]	−0.879** [0.359]
Nonfam CEO × Tenure			0.030** [0.014]		0.027** [0.014]
Nonfam CEO × Market				0.079* [0.043]	0.070 [0.043]
Tenure	−0.004 [0.008]	−0.004 [0.008]	−0.020** [0.011]	−0.004 [0.008]	−0.015 [0.099]
Market	0.032 [0.031]	0.035 [0.032]	0.034 [0.030]	−0.020 [0.043]	−0.012 [0.043]
Control variables	Control	Control	Control	Control	Control
_cons	5.632*** [1.317]	6.085*** [1.133]	6.149*** [1.298]	6.401*** [1.333]	6.575*** [1.335]
Wald χ^2^	98.98	102.58	105.04	98.55	102.33
*N*	1,330	1,330	1,330	1,330	1,330

*p < 0.1; **p < 0.05; and ***p < 0.01.

## Discussion

In family firms, the similarity in social status and competence have naturally lead to non-family managers making social comparisons between themselves and their counterparts. Compensation, as an initial symbol of a manager’s human capital value and social status (Main et al., 1993), has become an important object of social comparison. Non-family managers are likely to engage in social comparisons to compare their compensation with other managers to judge whether they get fair treatment (Collischon and Eberl, 2020). Hence, the fairness of compensation structure has been a prominent focus for non-family managers, and factors that could influence the pay dispersion have also attracted the attention of scholars. Family firms have either a family CEO or a non-family CEO, and their different characteristics alter non-family managers’ tendencies of social comparison. Despite being a basic and important corporate governance form in family firms, the effect of CEO identity has not received sufficient attention. Therefore, our work adopts a social comparison perspective to investigate how CEO identity influences non-family managers’ pay dispersion in family firms. In addition, the importance of CEO identity is mainly highlighted by society’s perceptions of competence and environmental informal rules. Our work also adopted CEO tenure and institutional environment as moderators to consider certain competence and environmental factors.

Using data from Chinese listed firms from 2009 to 2015, we hypothesize and find that that the presence of a non-family CEO can reduce the pay dispersion between the CEO and non-family managers. When non-family members held the CEO’s position, both the CEO and non-family managers have relatively similar social status and power, which renders non-family managers prone to compare with other non-family managers. To maintain the overall harmony of the top management team, the non-family CEOs will offer suggestions to the board of directors or the compensation committee to try to reduce the pay dispersion. Consequently, having a non-family CEO leads to a decreased level of pay dispersion between the CEO and non-family managers. By considering the moderating effects, since CEO tenure is a manifestation of ability and experience, a more capable CEO will be less likely to emphasize the importance of identity. CEOs with longer tenures are expected to obtain higher decision-making power and psychological ownership (Pittino et al., 2018), which would create a “power distance” between the CEO and non-family managers, and decreases their comparison tendency. In addition, environmental factors like institutional environment should also be considered. The more standardized and mature institutional environment leads to more attention to efficiency and fair competition, which will be less likely to emphasize the importance of CEO identity, thereby decreasing the comparison tendency. The results showed that both CEO tenure and institutional environment could moderate the relationship between CEO identity and pay dispersion, such that the negative effect of CEO identity on managerial pay dispersion weakens when CEO tenure increases and the institutional environment gets mature.

### Theoretical Implications

The current findings provide theoretical implications in three ways. First, our paper extends the recent research on the influencing factors of managerial pay dispersion in family firms and enhances the understanding of pay fairness in top management teams. Though the current research on pay dispersion influencing factors are mostly focused on the board level, our research begins by investigating the basic attributes of the top management teams, and highlighted CEO identity, a basic and important corporate governance form in family firms, as an important influencing factor of non-family managers’ pay dispersion. Under the framework of social comparison, our paper explores how CEO identity influences pay dispersion between CEO and non-family managers in family firms. Accordingly, we hypothesize and find that the presence of a non-family CEO can decrease the social comparison tendency between the CEO and non-family managers, and thereby lower the pay dispersion.

Second, in our work, CEO identity is first incorporated into the analytical framework of social comparison theory, which enriches the applicability of social comparison theory. Establishing a general view of the research on pay dispersion and social comparison theory, existing studies have shown that maintaining a reasonably low level of pay dispersion or maintaining pay harmony among top management teams has become a common outcome (Feldman et al., 2018). Even though maintaining pay fairness is of great significance for family firms, under the framework of social comparison theory, the current research does not indicate arrangements on how to maintain pay fairness or reduce pay dispersion. Hence, our paper highlights CEO identity to explore how CEO’s family or non-family identity influences the social comparison tendency of managers, and thereby influences pay dispersion. The results show that focusing on CEO identity in family firms could be an effective way to reduce pay dispersion between CEO and non-family managers. Therefore, this paper incorporates CEO identity into the analytical framework of social comparison theory and finds an effective way to deal with the pay dispersion, which enriches the applicability of social comparison theory.

Third, our paper makes an in-depth exploration of social comparison theory and finds some external factors that could affect individuals’ social comparison tendency. As a kind of individual subjective psychological feeling, social comparison can also be affected by some external factors, like individual competence and external informal rules. To specifically indicate them, our paper highlights CEO tenure and institutional environment as moderators. At the same time, the importance of CEO identity also depends on society’s perception of competence and external informal rules. For competence factor, CEO tenure is an important indicator to identify CEO’s competence and experience (Chen et al., 2019), and higher competence leads to a reduced emphasis on identity. A more capable CEO will be less likely to emphasize the importance of identity, which will decrease the comparison tendency among non-family managers. Environmental informal factors like institutional environment should also be considered. A more standardized institutional environment led to more attention on efficiency and fair competition, which will be less likely to emphasize CEO identity, thereby decreasing the comparison tendency among managers. Consequently, our paper makes an in-depth exploration of social comparison theory and finds some external factors that could affect the social comparison tendency.

### Practical Implications

First, recruitment of capable non-family managers is inevitable when family firms pursue a formal management structure and sustainable development. In the context of family firm professionalization, retention and motivation of professional managers are some of the most important issues. The results of this study show that non-family managers are highly sensitive to the fairness of compensation in family firms. Therefore, when designing the compensation mechanism for non-family managers, family firms should not only pay attention to the compensation level but also consider the fairness of the compensation to facilitate the stability of these members.

Second, though the compensation arrangement for the top management teams is generally determined by the board of directors or the compensation committee, our findings consider that CEOs also have the ability and motivation to determine top managers’ compensation, which will impact managerial pay dispersion. Therefore, the fairness of compensation among non-family managers should also consider the impact of CEOs when setting the compensation for non-family managers.

### Limitations and Future Research

Our study has several limitations that generate opportunities for future research. First, the data on managerial pay dispersion were only related to cash compensation and ignored other compensation forms like stock-based compensation. The reasons are as follows: first, since the phenomenon of managers “holding zero stock” generally exists in China, there are a certain number of missing values of stock-based compensation, which would have an impact on our regression results. Second, to keep family control, family firm owners are reluctant to offer stock-based compensation for non-family managers (Michiels et al., 2013). Third, the fluctuation of stock price will lead to fluctuations in managers’ stock-based compensation, which will greatly increase the difficulty of calculating the pay dispersion, and reduce the accuracy of data. However, since option incentives and dividends are gradually being adopted in managerial incentive systems in Chinese listed firms in these years, we will try to focus on diverse compensation types in future research.

Second, for the moderating effects, we only included factors of CEO tenure and institutional environment, but factors related to family firms’ attributes were ignored. The reasons are as follows. Moderating variables are used to highlight or dilute the impact of CEO identity on pay dispersion. However, since CEOs are the core leader of the top management teams, some variables related to family firms like family ownership or second-generation involvement have little influence on the decision-making power exerted by CEO identity, and so has little influence on the relationship between CEO identity and pay dispersion. In addition, the empirical results show that multicollinearity exists between CEO identity and these variables. Hence, our future research will try to explore some other variables related to family firms. These variables could be included not only in the context of family firms but also in the influence of CEO identity and managerial compensation, which remain unexplored.

## Conclusion

This research is primarily to study how CEO identity influences the pay dispersion between CEO and non-family managers in family firms, and the moderating roles of CEO tenure and institutional environment. The findings of this study contribute to the research on influencing factors of managerial pay dispersion by highlighting CEO identity as an important factor to influence non-family managers’ pay dispersion in family firms. Prior studies have considered the influencing factors at the board level, whereas our study considered these effects in one of the basic top management team attributes: CEO identity in family firms. The research also enriches the applicability of social comparison theory by bringing CEO identity into the analytical framework of social comparison theory. The findings also suggest that to retain non-family managers’ human and social capital, it is important to focus on fairness when setting compensations structures for these managers. Furthermore, the fairness of compensation should not ignore the role of CEOs. The study also has several limitations that generate opportunities for future research. Due to data availability and accuracy, our paper does not consider stock-based compensation when measuring pay dispersion. Also, some family firms attributes are ignored. Future research should further focus on diverse compensation forms and explore more family firm attributes factors.

## Data Availability Statement

The datasets presented in this study can be found in online repositories. The names of the repository/repositories and accession number(s) can be found below: https://www.gtarsc.com/.

## Author Contributions

WZ: collected and analyzed the data and drafted the manuscript. LC: reviewed the manuscript and revised the manuscript. J-aZ: designed the research protocol and contributed in literature review. All authors have read and agreed to the published version of the manuscript.

## Conflict of Interest

The authors declare that the research was conducted in the absence of any commercial or financial relationships that could be construed as a potential conflict of interest.

## Publisher’s Note

All claims expressed in this article are solely those of the authors and do not necessarily represent those of their affiliated organizations, or those of the publisher, the editors and the reviewers. Any product that may be evaluated in this article, or claim that may be made by its manufacturer, is not guaranteed or endorsed by the publisher.
